# Global Transcriptome Characterization and Assembly of the Thermophilic Ascomycete *Chaetomium thermophilum*

**DOI:** 10.3390/genes12101549

**Published:** 2021-09-29

**Authors:** Amit Singh, Géza Schermann, Sven Reislöhner, Nikola Kellner, Ed Hurt, Michael Brunner

**Affiliations:** Heidelberg University Biochemistry Center (BZH), Im Neuenheimer Feld 328, D-69120 Heidelberg, Germany; amit.singh@bzh.uni-heidelberg.de (A.S.); Geza.Schermann@medma.uni-heidelberg.de (G.S.); sven.reisloehner@bzh.uni-heidelberg.de (S.R.); nikola.kellner@bzh.uni-heidelberg.de (N.K.); ed.hurt@bzh.uni-heidelberg.de (E.H.)

**Keywords:** genome-wide annotation, *Chaetomium thermophilum*, transcriptome assembly, R package, Enzyme Commission number, Gene Ontology, industrial application, novel genes

## Abstract

A correct genome annotation is fundamental for research in the field of molecular and structural biology. The annotation of the reference genome of *Chaetomium thermophilum* has been reported previously, but it is essentially limited to open reading frames (ORFs) of protein coding genes and contains only a few noncoding transcripts. In this study, we identified and annotated full-length transcripts of *C. thermophilum* by deep RNA sequencing. We annotated 7044 coding genes and 4567 noncoding genes. Astonishingly, 23% of the coding genes are alternatively spliced. We identified 679 novel coding genes as well as 2878 novel noncoding genes and corrected the structural organization of more than 50% of the previously annotated genes. Furthermore, we substantially extended the Gene Ontology (GO) and Enzyme Commission (EC) lists, which provide comprehensive search tools for potential industrial applications and basic research. The identified novel transcripts and improved annotation will help to understand the gene regulatory landscape in *C. thermophilum.* The analysis pipeline developed here can be used to build transcriptome assemblies and identify coding and noncoding RNAs of other species.

## 1. Introduction

*Chaetomium thermophilum* is a thermophilic filamentous ascomycete, with the ability to grow at 50–52 °C. It lives saprophytically and thrives on decomposing plant material [1]. Its lignocellulolytic lifestyle produces different lignocellulolytic thermostable enzymes, such as cellulase, xylanase, laccase, chitinases, and proteases [2,3,4,5,6]. The thermostability of these enzymes makes *C. thermophilum* a model organism of choice in various biotechnological, pharmaceutical, and food processing industries. In the past decade, *C. thermophilum* has attracted attention to different applications such as starch degradation, hydrolysis of cellulose for bioethanol production, as well as other applications requiring enzymatic activities at higher temperatures [6,7,8,9,10,11,12,13,14]. Additionally, owing to the thermostability of its proteins, the structures of many *C. thermophilum* proteins and protein assemblies have been solved with high resolution in various crystallization and cryo-electron microscopy studies, which improved our understanding of the structural organization and function of higher order protein complexes. These include the Crm1 export factor, the splicing factor Cwc27, mRNA export factor Mex67-Mtr2, the FACT complex, the eukaryotic RAC chaperone, the nuclear pore Nsp1-channel complex, and the 90 S pre-ribosomal complex [15,16,17,18,19,20,21,22]. The initial annotation of the genome of *C. thermophilum* [23] was substantially improved [24]. It has a size of 28.3 MB and was assembled into 20 scaffolds containing 7165 protein coding and 387 noncoding genes. Despite recent advances in sequencing technologies [25,26,27,28], the *C. thermophilum* genome annotation has not been substantially improved as it lacks proper annotation of untranslated regions (UTRs), and the majority of intron-exon structures are computationally predicted rather than experimentally determined. Given the large increase in the number of genomic studies on *C. thermophilum,* a comprehensive genome annotation will be helpful for further functional, structural, proteomic, genomic, and transcriptomic analyses. In this study, we present an improved annotation of the *C. thermophilum* genome based on deep RNA sequencing and establish pipeline tools for the analysis of sequencing data. Our annotation identified 7044 expressed protein-coding genes and 4567 long noncoding RNAs (lncRNAs). Moreover, we detected UTRs and intron-exon boundaries as well as transcript isoforms. Sequence homology studies revealed that *C. thermophilum* and *Thermothelomyces thermophila* share close sequence similarity of coding transcripts. Downstream analysis of genomic and transcriptomic sequence data is widely used to predict gene function, identify biomarkers, and group and classify gene expression patterns. Therefore, we present an extended Gene Ontology (GO) and Enzyme Commission (EC) numbers associated with the protein-coding genes of *C. thermophilum*.

## 2. Materials and Methods

### 2.1. RNA Isolation and Sequencing

A *C. thermophilum* wild type strain was received from DSMZ, Braunschweig, Germany (No. 1495). The mycelium was harvested from an overnight grown CCM plate [29] and subsequently cultured in liquid growth medium (0.5 g NaCl, 0.65 g K_2_HPO_4_·3H_2_O, 0.5 g MgSO_4_·7H_2_O, 0.01 g Fe (III)-sulfatehydrate, 10 g D-glucose, and 1 g each of peptone and yeast extract per liter H_2_O, pH 7.0) in 250 mL Erlenmeyer flasks at 52 °C and 110 rpm for six hours. The sieved and dried biomass was ground to a fine powder in liquid nitrogen. Then, 100 mg of mycelial powder from three independent biological replicates was used for total RNA-extraction using the SV total RNA isolation system (Promega). The libraries were prepared with the NEBNext Ultra II Directional RNA Preparation Kit for Illumina in combination with NEBNext PolyA selection Module, plus the NEBNext Multiplex Oligos for Illumina, and single-end sequencing was performed by the CellNetworks Deep Sequencing Core Facility (Heidelberg, Germany) on an Illumina NextSeq 500 platform.

### 2.2. RNA Sequencing Data Analysis

**Figure 1:**
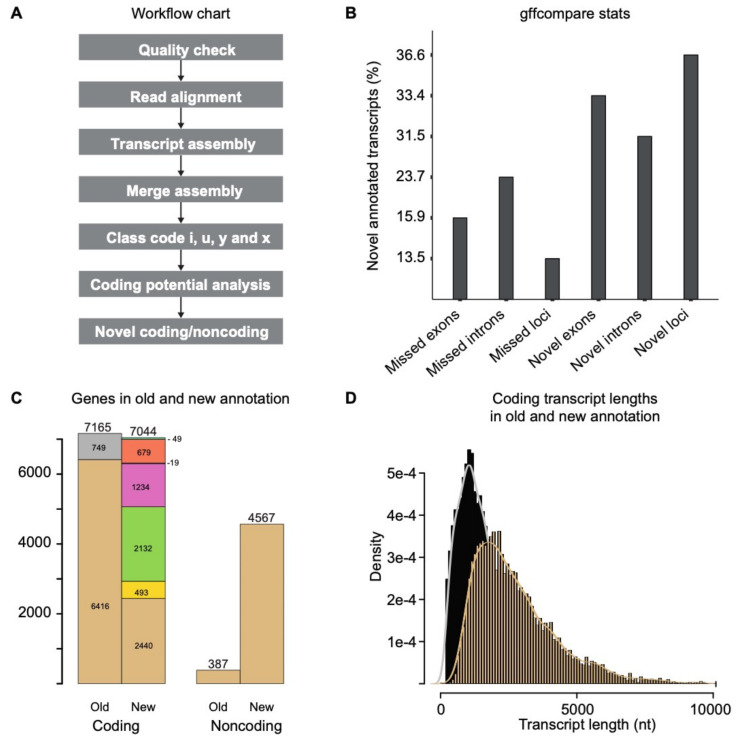
Transcriptome-based annotation of the genome of *C. thermophilum*. (**A**) Schematic overview of the analysis pipeline. (**B**) Fraction of newly annotated transcript that differs from the previous annotation by the indicated *gffcompare* feature. Note that the transcripts add up to more than 100%. (**C**) Stack bar graph comparison of the number of genes of the old and new annotations. Differences are indicated by colored boxes. Coding genes, old annotation: the stacked grey bar represents the number of coding genes (749) that were not expressed (detected) in our conditions. Coding genes, new annotation: Beige: genes without change in the intron structure (*n* = 2440). Yellow: genes where at least one isoform has a novel splicing variant (*n* = 493). Green: genes where all transcripts have at least one intron-exon junction different than in the previous annotation (*n* = 2132). Pink; genes where all junctions are different (*n* = 1234). Blue; genes that are flipped (opposite strand with same or similar splice junctions) (*n* = 19). Orange; completely novel genes (*n* = 679). Aquamarine: uncategorized genes (*n* = 49). (**D**) Histogram representing the lengths of coding transcripts from the new annotation (beige) compared with the previous annotation of ORFs (black). Cutoff at 10,000 nt.

The quality assessment of raw sequence data was performed by FastQC (Version: FastQC 0.11.5) (http://www.bioinformatics.babraham.ac.uk/projects/fastqc/, accessed on 10 April 2018). No samples were discarded from the analysis. The *C. thermophilum* reference genome and gene annotation files were downloaded from the Ensemble genome browser (version 2.8) and a pipeline was developed to annotate UTR regions and to identify putative novel transcripts (Figure 1A). The raw reads were mapped to the *C. thermophilum* genome using HISAT2 with the following parameters (Version: 2.1.0; [hisat2 -p 8 -x -max-intronlen 2000 -dta-U]) [30]. For each sample, the mapped reads from HISAT2 were assembled separately using StringTie with parameter settings (Version: 1.3.3b; [stringtie -o -m 50 -p 8 -j 3 -c 5 -g 15]) [31]. The multiple transcript assembly files from the different samples were used together to produce a distinctive transcriptome set using gffcompare with parameter settings (Version: v0.10.1; [gffcompare-merge -K -o gffcomp -i]) [25]. Based on the previous assembly results, transcripts shorter than 200 nt were excluded to identify transcripts from the merged transcript assembly. According to gffcompare, class codes “i”, “u”, ”y”, and “x” were considered novel transcriptional loci. The coding potential calculator (CPC2) was used to evaluate the coding potential of all transcripts [32].

### 2.3. Sequence Conservation and GO Annotation

The sequence conservation analysis was performed using dc-mega BLAST (Version: 2.7.1+) [33]. All coding and noncoding transcripts including the identified novel transcript sequences were used for this analysis. *Sordaria macrospora* (NCBI taxid-5147), *Neurospora crassa* (NCBI taxid-5141), *Aspergillus niger* (NCBI taxid-5061), *Saccharomyces cerevisiae* (NCBI taxid-4932), *Takifugu rubripes* (NCBI taxid-31033), and *Thermothelomyces thermophila* (NCBI taxid-78579) were chosen to study the sequence similarity analysis using BLAST (E value, 1 × 10^−3^). phyloT (https://phylot.biobyte.de, accessed on 24 April 2018) was used for the construction and visualization of a phylogenetic tree of the species mentioned above. Additionally, the functional annotation of *C. thermophilum* transcripts was analyzed using Blast2GO [Version 5.1.1] [34], as described in the manual. The annotated GO terms from *Thermothelomyces thermophila*, *Neurospora crassa*, and *Sordaria macrospora* were used as an input for the Blast2GO analysis, based on local blastx. Enzyme Commission numbers were obtained using the same method. The data visualization was carried out using R (Version 3.3.3) [35].

### 2.4. Isoform Annotation

To create the isoform annotation, 9772 coding transcripts were analyzed. The longest non-reverse ORF, in the search order of [blastx-hit-frame1-frame2-frame3], using ATG as start codon and an obligatory stop codon was obtained from Blast2GO. Sequences were grouped by the transcription loci tags pyfaidx python package [36]. All groups were aligned [-output=aln] and similarities were calculated [-otherpg seqreformat -output sim] using the T-Coffee software [37]. Pairwise similarity scores formed two distinct groups. Based on this, the score cutoff level was set at 49 for designating isoforms. All low scoring accepted hits (score 49–60) were manually checked and visualized for correctness. The accepted protein sequence pairs were merged into isoform groups by connectivity calculation in R programming language. Noncoding transcripts were grouped by overlapping features. Software packages, the webpage, and its usage are summarized in Appendix A (Table A1).

## 3. Results

### 3.1. Transcripts Reassembly and Identification of Novel Transcripts

The single-end RNA sequencing data of *C. thermophilum* were obtained in triplicates with a read length of 85 bp. A schematic overview of the analysis pipeline is shown in (Figure 1A). We performed short read gapped alignment using HISAT2 [30] and recovered more than 95% of mapped reads, as shown in Appendix A (Table A2). We used StringTie [31] to de novo assemble the three samples separately. The assembled transcript files from these three samples were merged into a combined set of transcripts using the gffcompare utility provided by Cufflinks. After manual curation of 17 transcripts and filtering the transcript length (>200 nt), a total of 15,363 reliable transcripts were obtained. A comparison of these transcripts (Gffcompare statistics) compared with the previously annotated genes is shown in Figure 1B. In total, the transcripts were assigned to 7044 coding genes represented by 9772 transcripts and transcript isoforms, and 4567 noncoding genes represented by 5591 transcripts and transcript isoforms (Figure 1C). The transcripts annotated class codes are listed in Appendix A (Table A2). Transcripts annotated to gffcompare classes u (no overlap, *n* = 2744), x (opposite strand, *n* = 1754), i (contained in reference intron, *n* = 28), and y (contains a reference gene within intron, 5 transcripts) are called novel transcripts in our analysis. By these criteria, we identified 679 novel coding genes represented by 892 transcripts and isoforms, as well as 2878 novel noncoding genes represented by 3639 transcripts and isoforms. Further, 749 genes that were classified as coding in the previous annotation were not detected/expressed under our experimental condition (Table S1). These non-expressed putative genes show low conservation in related fungi (below 40%), suggesting that this group may contain falsely annotated genes. Figure 1D represents the increased lengths of the newly annotated transcripts compared with the earlier annotation, which did not include UTRs. Finally, we created a gene annotation database containing all the above information in a TxDb framework in the R package [38] for *C. thermophilum*. The gene annotation in gene transfer format (gtf) as well as in Microsoft Excel format can be found in Table S2 and Table S3, respectively.

### 3.2. Annotation of the Novel Noncoding RNAs in C. Thermophilum

**Figure 2:**
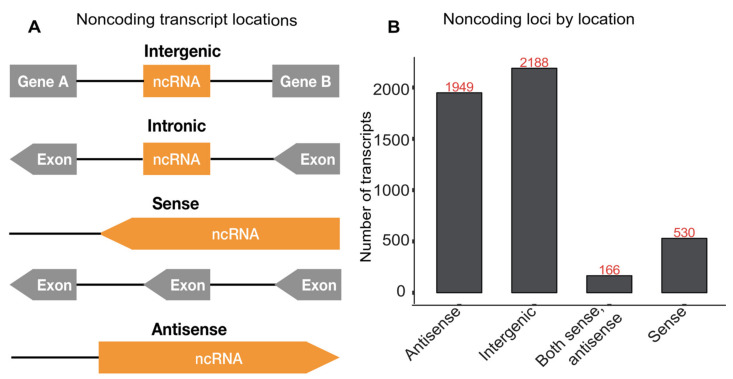
Location of noncoding transcripts. (**A**) Schematic representation of possible locations of noncoding transcripts relative to coding genes. (**B**) Bar graph showing the number of noncoding transcripts at the indicated genomic locations.

We identified 5591 noncoding RNA transcripts based on the CPC2 analysis. These include highly expressed contaminant RNA species such as ribosomal RNAs, t-RNAs, snoRNAs, RNase RNAs, and snRNAs. The remaining noncoding transcripts were classified as intronic, intergenic, sense overlap with coding gene, and antisense based on association with annotated protein-coding genes (Figure 2A). In total, we identified 2188 lincRNA genes as intergenic, 1949 antisense genes, and 530 genes overlapping in the sense direction. Moreover, 166 genes are in both sense and antisense; these could represent truncated mRNAs or functional RNAs involved in gene regulation (Figure 2B).

### 3.3. Sequence Conservation and Functional Annotation

**Figure 3:**
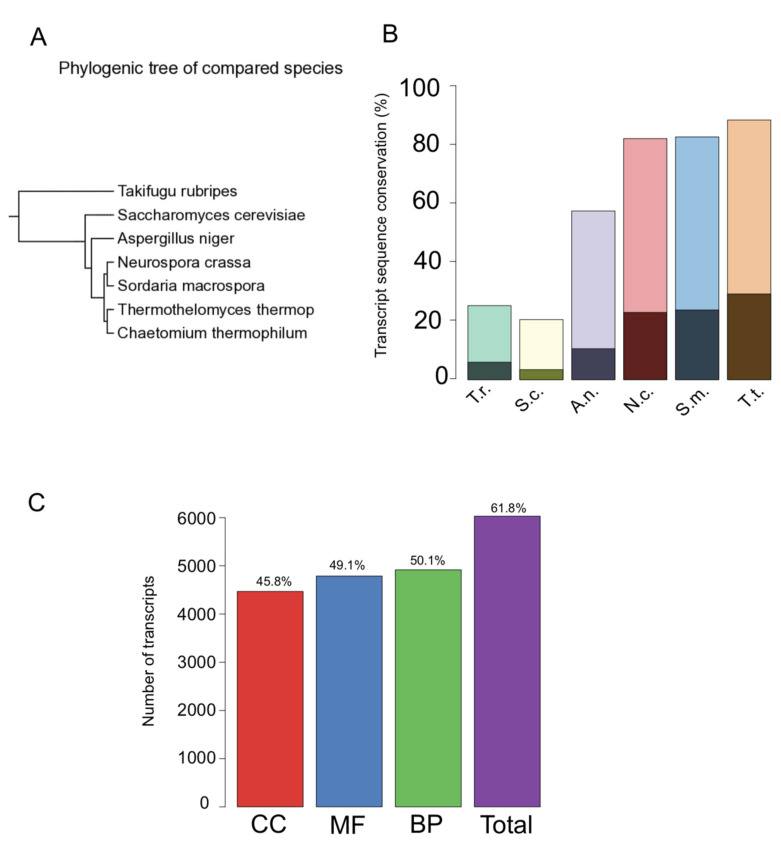
Phylogenetic conservation of coding and noncoding *C. chaetomium* transcripts. (**A**) Phylogenic tree of six indicated species. (**B**) Double bar graph representing the sequence similarity of *C. thermophilum* transcripts with six indicated species. Light bars correspond to the percentage of similar coding transcripts. The dark bars in front correspond to the percentage of noncoding transcripts. (**C**) Number of transcripts associated with the GO annotations “biological process” (BP), “molecular function” (MF), and “cellular compartment” (CC). The right bar indicates the total number of transcripts associated with at least one GO term. The percentage of transcripts associated with the respective GO terms is indicated. Note that a given transcript can be associated with more than one GO term.

To assess the conservation of coding and noncoding *C. thermophilum* genes, we constructed a phylogenetic tree including six other species. The analysis indicates that *C. thermophilum* is closely related to *Thermothelomyces thermophila* and more distantly to *Neurospora crassa* and *Sordaria macrospora* (Figure 3A). A local dc-megaBLAST similarity search was carried out for the newly annotated coding, anti-sense, and lincRNAs of *C. thermophilum*. We found that about 90% of the coding and about 30% of the noncoding sequences share significant similarity with *Thermothelomyces thermophila* (Figure 3B). Furthermore, about 80% of the coding and 25% of the noncoding sequence of both *Sordaria macrospora* and *Neurospora crassa* share similarity with *C. thermophilum* (Figure 3B).

Gene Ontology (GO) analysis facilitates the functional annotation of genes. We, therefore, used Blast2GO to associate the transcripts with functional annotation. Altogether, we found 4283 GO terms. With these, we could annotate 6031 coding transcripts (corresponding to 4336 genes) (Figure 3C). A total of 4468 transcripts (3280 genes) belong to cellular component (CC), 4787 transcripts (3445 genes) belong to molecular function (MF), and 4916 transcripts (3572 genes) belong to biological process (BP). All GO terms are listed in Table S3. The number of transcripts associated with the top 10 GO-slim terms for each category (MF, CC, BP) is shown in Figure S1. Further, we created a GO annotation R package for *C. thermophilum* using the function makeOrgPackage [38]. To facilitate functional gene finding for potential industrial applications, we additionally retrieved Enzyme Commission numbers (E.C.) from the Blast2GO analysis. We could associate 1802 coding transcripts (corresponding to 1366 genes) with 643 E.C. numbers (Table S3). The main E.C. class distribution is shown in Figure S2.

## 4. Discussion

*C. thermophilum* belongs to the group of filamentous fungi that are an economically important, as it developed not only into a relevant resource in pharmaceutical and food processing industries, as well as second generation biofuel production, but also became a scientifically important model organism in basic research. The structural biochemistry community highly appreciates *C. thermophilum* for the analysis of large and/or dynamic protein complexes, particularly in the field of ribosome biogenesis, where its superior protein stability gave rise to unprecedented subatomic cryo-electron microscopy 3D structures [12,13,14,22,39,40,41,42,43,44,45,46,47]. To facilitate further intriguing research on *C. thermophilum* for challenging protein biochemistry studies and biotechnological applications, we greatly improved the understanding of its genetic architecture with a global deep sequencing approach. Additionally, an analysis pipeline was developed to characterize the transcriptome of *C. thermophilum*. The main aim of the study was to provide an improved gene annotation, containing high fidelity coding and noncoding transcripts and isoforms with both 3′ and 5′ UTRs. We evaluated all transcript class codes from our transcriptome assembly and compared them with the previous annotation. We observed substantial discrepancies with the previous annotation of *C. thermophilum*, as only 2935 coding and 143 noncoding transcripts displayed a complete intron match (class code “=“). The majority of coding transcripts showed at least one intron mismatch compared with the old annotation (class code “j”). We observed that 749 coding and 254 noncoding transcripts of the previous annotation were not detected (no overlaps with any identified transcript) in our analysis. Moreover, 104 of these genes were potentially expressed in other growth conditions >100 read counts (unpublished RNA sequencing data), while the remaining genes were not expressed. Furthermore, most of these genes had no homology in related species, suggesting that they might have been wrongly annotated. These unexpressed transcripts are listed in Table S1.

Our transcript assembly revealed that 1640 genes express at least 2–3 transcript isoforms (4368 transcripts, 44.7%), indicating rather complex alternative splicing in *C. thermophilum*. A handful of genes may express even higher numbers of isoforms. However, owing to theoretical limitations in the analysis of the single-end sequencing data, such potentially complex isoforms cannot be reliably predicted. Here, we present all potential transcript isoforms, and the corresponding predicted protein sequences are listed in Table S4 in text file format. Moreover, our analysis revealed a surprisingly high number of noncoding transcripts, both lincRNA and antisense RNAs. Together with the alternative splicing of coding genes, our data suggest a complex transcriptional network of *C. thermophilum*. Comparative sequence analysis uncovered that *C. thermophilum* and *Thermothelomyces thermophila* share 90% genome sequence similarity, as shown in Figure 3B, which is likely to reflect related functions, potentially associated with their thermophilic lifestyles. Functional annotation through Gene Ontology (GO) associations facilitate the interpretation of genomic and transcriptomic sequence data. Functional annotation achieved by the integration of several databases such as KEGG [48], UniProt [49], InterPro [50], Pfam [51], NCBI [52], SEED [53], ConsensusPathDB [54], Reactome [55], and structural annotation tool [56] may help address this question. Our Blast2GO analysis substantially expands the GO term annotations in *C. thermophilum*. Hence, our annotation and transcript assembly open opportunities for the systematic functional analysis of *C. thermophilum* proteins. An example would be a known protein fragment of a laccase enzyme from *C. thermophilum* (Uniprot ID: Q692I0) that was so far not annotated in the genome and the full protein sequence was unknown. We revealed that the gene was expressed in our conditions and have annotated the complete transcript and protein sequence (Gene001724). *C. thermophilum* genes responsible for the formation of conidia are another example of an extended functional annotation. The reproduction cycle of *C. thermophilum* is not well understood, and previously only a single gene had been associated with the formation of asexual spores (conidia). Our GO term annotation revealed that several additional genes, such as Gene000283, Gene003743, Gene006754, and Gene006902, are associated with the conidiation process (GO:0048315, GO:0030437, GO:0030435).

Our analysis is solely based on a single growth condition with single-end RNA seq data. Hence, different growth conditions, as well as paired-end sequencing data, could further improve the gene annotation. It has been observed previously that *C. thermophilum* is extremely prone to RNA degradation. Therefore, an improved RNA isolation procedure could decrease the number of truncated transcripts and might further advance the analysis of the *C. thermophilum* transcriptome in the future. In summary, we detected a large number of novel coding and noncoding transcripts and discovered an abundance of alternative splicing in *C. thermophilum*. Thus, our study provides useful resources for functional genomics and proteomics research on *C. thermophilum* and facilitates the analysis of biological and biochemical processes. Moreover, our analysis pipeline could in principle also be used to annotate the genomes of other organisms.

## Data Availability

The GO Term and gene annotation R packages of *C. thermophilum* are available in the following link: https://github.com/amit-singh25/Chaetomium-thermophilum, accessed on 17 August 2021.

## Supplementary Materials

The following are available online at https://www.mdpi.com/article/10.3390/genes12101549/s1. Figure S1: Bar graph of top abundant GO slim terms obtained from Blast2GO analysis. GO terms and the corresponding number of transcripts are indicated. Color code: yellow = “cellular component”; green = “biological process”; blue = “molecular function”; Figure S2: (A) Bar graph showing the number of transcripts corresponding to the six major enzyme class codes. (B) Bar graphs 1–6 show the number of transcripts corresponding to the indicated enzyme subclass codes; Table S1: Excel file of 749 coding genes of the old annotation that were not detected in our analysis; Table S2: Complete gene list with essential attributes/annotation feature in gtf file format, which can be used in further genomics and transcriptomics analyses; Table S3: Genes associated with GO annotation and E.C. numbers; Table S4: List of translated protein sequences of all ORFs ≥50 aa containing start and stop codons.

## Accession Numbers

The RNA seq and genome annotation data are deposited in the Gene Expression Omnibus (GEO) database under accession no GSE116834.

## Appendix A

**Table A1:** (**A**): RNA-seq data alignment results for reads of different samples. (**B**): Different classes of assembled transcripts.

**(A)**		
**Sample Name**	**Input Read**	**Overall Alignment Rate**
G1	69350606	95.94%
G2	73230226	95.87%
G3	59976502	95.83%
**(B)**		
**Class Code**	**Description**	**Total Annotation**
=	Complete match of intron chain	3078
c	Contained in reference (and intron isoform compatible)	323
k	Containment of reference (reverse containment)	0
j	At least one splice junction match	4234
e	At single exon, overlapping intron a possibly pre-mRNA fragment (un spliced intron)	420
o	Other same strand overlap with reference exons	1896
s	Intron match on the opposite strand (likely a mapping error)	158
x	Exonic overlap on the opposite strand (like ‘o’ or ‘e’, but on the opposite strand)	1754
i	Fully contained in a reference intron	28
y	Contains a reference within is intron(s)	5
p	Possible polymerase run-on (no actual overlap)	706
r	Repeat (at least 50% bases soft masked)	0
u	None of the above (unknown, intergenic)	2744

**Table A2:** RNA-seq data alignment results for three different samples.

Software	Usage	Parameter Settings/Webpage
Ensemble genome (version 2.8)	*C. thermophilum* reference genome used for the analysis	https://fungi.ensembl.org, accessed on 17 August 2021
FastQC (Version: 0.11.5)	The data quality assessment of raw sequence data	http://www.bioinformatics. babraham.ac.uk/projects/fastqc/, accessed on 17 August 2021
HISAT2 (Version: 1.3.3b)	The raw reads were mapped to *C. thermophilum* reference genome	[hisat2 -p 8 -x -max-intronlen 2000 -dta-U])
Stringtie (v1.3.4 release)	The mapped reads from HISAT2 for each sample were assembled separately	[stringtie -o -m 50 -p 8 -j 3 -c 5 -g 15])
GffCompare (Version: v0.10.1)	The program used to compare, merge, annotate, and estimate accuracy “query” files, when compared with a reference annotation	gffcompare-merge -K -o gffcomp -i
CPC2	The CPC2 calculate the coding or noncoding of the transcript	CPC2.py -i. Input.fasta -o output.txt
dc-mega BLAST (Version: 2.7.1+)	Sequence conservation analysis between different species	https://blast.ncbi.nlm.nih.gov, accessed on 17 August 2021
Blast2GO (Version 5.1.1)	Functional annotation such as GO term and EC number are extracted from the software	http://docs.blast2go.com/, accessed on 17 August 2021
blastx.	Finds regions of local similarity between sequences	https://blast.ncbi.nlm.nih.gov/, accessed on 17 August 2021
T-Coffee software	Pairwise similarity score are calculated	http://tcoffee.crg.cat/, accessed on 17 August 2021
phyloT	Visualization of phylogenetic tree different species	https://phylot.biobyte.de, accessed on 17 August 2021
pyfaidx python package	Allowing for fast random access to any subsequence in the indexed FASTA file	https://pypi.org/project/pyfaidx/, accessed on 17 August 2021
R (Version 3.3.3)	Creating figures and GO annotation package	http://www.R-project.org/, accessed on 17 August 2021
