# Supplementary material for: Global Transcriptome Characterization and Assembly of the Thermophilic Ascomycete Chaetomium thermophilum
Source: Genes (Basel). 2021 Sep 29;12(10):1549. doi: 10.3390/genes12101549 (PMC8535861; doi:10.3390/genes12101549)
Supplement: Supplementary file 1 [file genes-12-01549-s001.zip › Table-1.pdf]

Table- 1A: RNA-seq data alignment results for reads of different samples.

Sample name	Input read	overall alignment rate
G1	69350606	95.94%
G2	73230226	95.87%
G3	59976502	95.83%

Table- 1B: Different classes of assembled transcripts

Class code	Description	Total annotation
=	Complete match of intron chain	3078
c	Contained in reference (and intron isoform compatible)	323
k	containment of reference (reverse containment)	0
j	At least one splice junction match	4234
e	At Single exon, overlapping intron a possibly pre-mRNA fragment (un spliced intron)	420
o	Other same strand overlap with reference exons	1896
s	Intron match on the opposite strand (likely a mapping error)	158
x	Exonic overlap on the opposite strand (like ,'o' or 'e' but on the opposite strand)	1754
i	fully contained in a reference intron	28
y	Contains a reference within is intron(s)	5
p	Possible polymerase run-on (no actual overlap)	706
r	repeat (at least 50% bases soft masked)	0
u	none of the above (unknown, intergenic)	2744
