# Supplementary figures and images for: Global Transcriptome Characterization and Assembly of the Thermophilic Ascomycete Chaetomium thermophilum
Source: Genes (Basel). 2021 Sep 29;12(10):1549. doi: 10.3390/genes12101549 (PMC8535861; doi:10.3390/genes12101549)

# Most abundant Go-slim terms

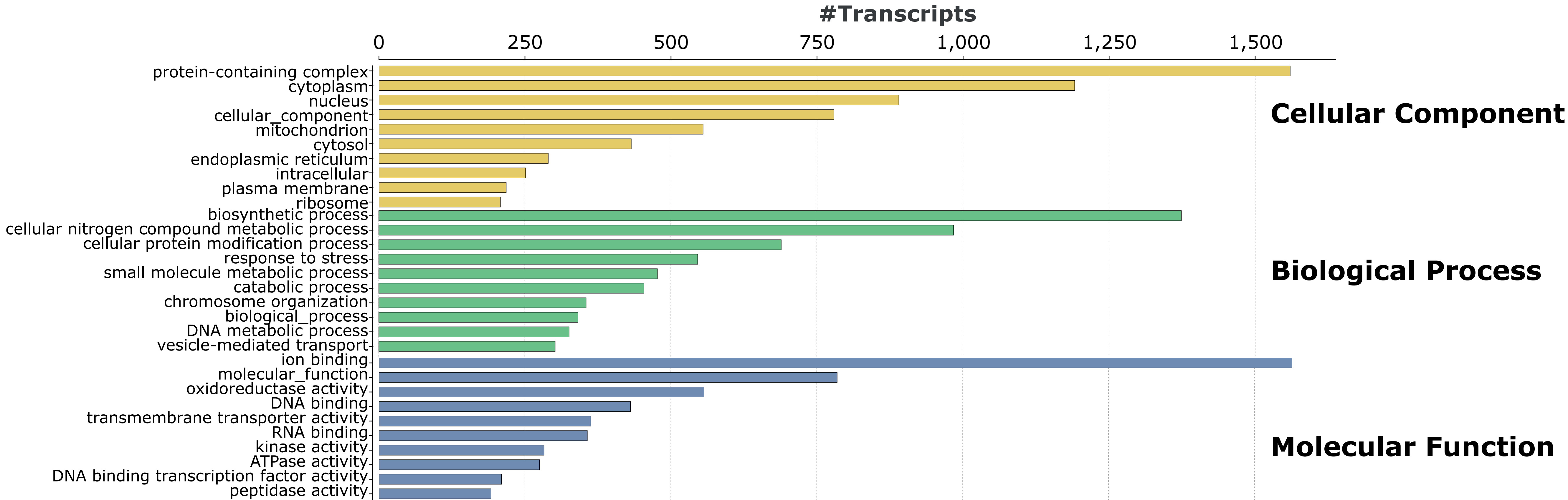

Supplement: Supplementary file 1 [file genes-12-01549-s001.zip › Supplementary Figure-1.pdf]

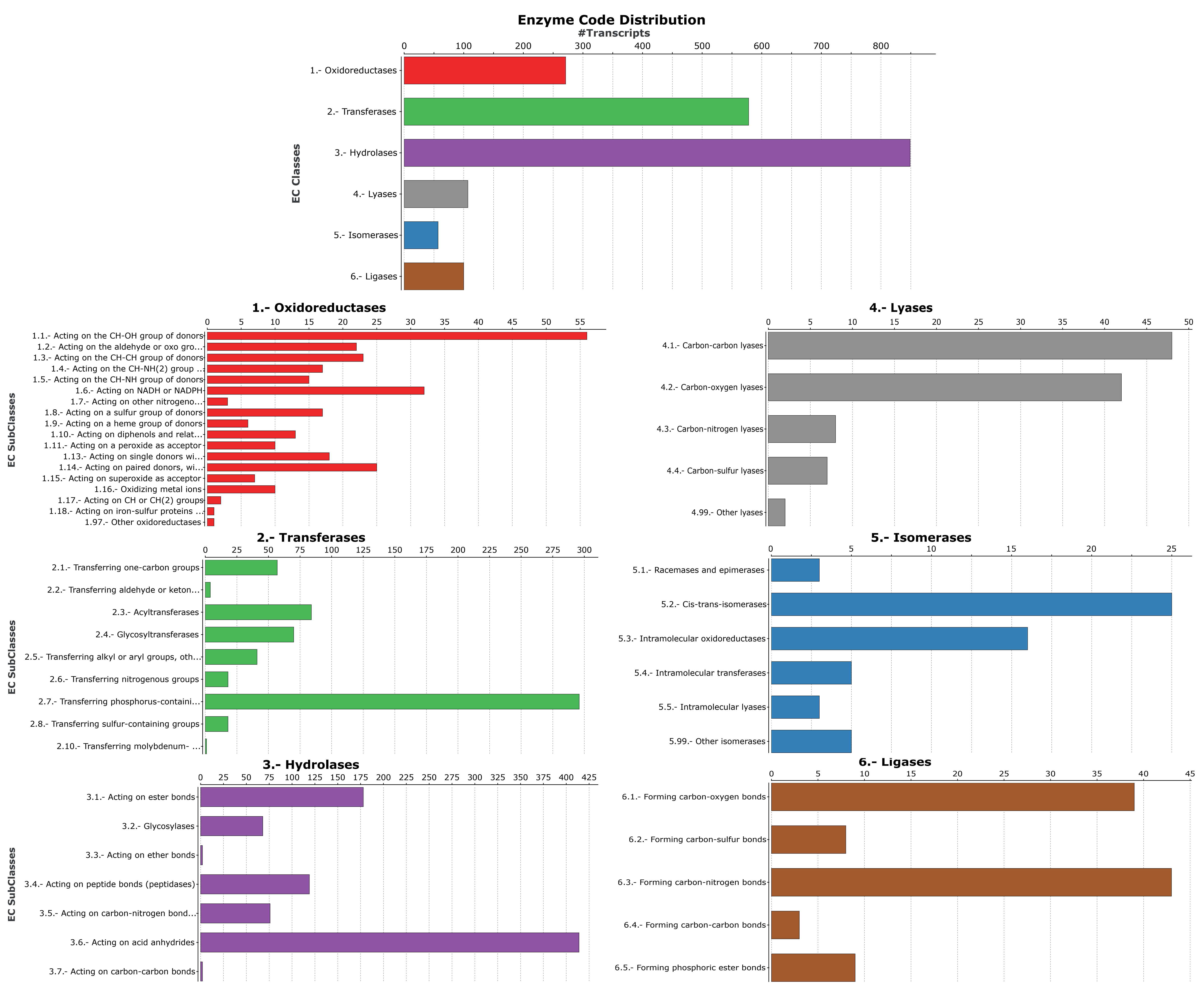

Supplement: Supplementary file 1 [file genes-12-01549-s001.zip › Supplementary Figure-2.pdf]
